# Novel Multi-Responsive Hyperbranched Polyelectrolyte Polyplexes as Potential Gene Delivery Vectors

**DOI:** 10.3390/pharmaceutics15061627

**Published:** 2023-05-30

**Authors:** Dimitrios Selianitis, Hector Katifelis, Maria Gazouli, Stergios Pispas

**Affiliations:** 1 Theoretical and Physical Chemistry Institute, National Hellenic Research Foundation, 48 Vassileos Constantinou Avenue, 11635 Athens, Greece; dselianitis@eie.gr; 2 Laboratory of Biology, Department of Basic Medical Science, School of Medicine, National and Kapodistrian University of Athens, 11527 Athens, Greece; katifel@med.uoa.gr (H.K.); mgazouli@med.uoa.gr (M.G.)

**Keywords:** hyperbranched polyelectrolyte copolymers, pH/thermo-sensitive, polyplexes, short DNA, MTT assay, gene delivery

## Abstract

In this work, we investigate the complexation behavior of poly(oligo(ethylene glycol)methyl methacrylate)-co-poly(2-(diisopropylamino)ethyl methacrylate), P(OEGMA-co-DIPAEMA), hyperbranched polyelectrolyte copolymers, synthesized by reversible addition fragmentation chain transfer (RAFT) polymerization, with short-linear DNA molecules. The synthesized hyperbranched copolymers (HBC), having a different chemical composition, are prepared in order to study their ability to bind with a linear nucleic acid at various N/P ratios (amine over phosphate groups). Specifically, the three pH and thermo-responsive P(OEGMA-co-DIPAEMA) hyperbranched copolymers were able to form polyplexes with DNA, with dimensions in the nanoscale. Using several physicochemical methods, such as dynamic and electrophoretic light scattering (DLS, ELS), as well as fluorescence spectroscopy (FS), the complexation process and the properties of formed polyplexes were explored in response to physical and chemical stimuli such as temperature, pH, and ionic strength. The mass and the size of polyplexes are shown to be affected by the hydrophobicity of the copolymer utilized each time, as well as the N/P ratio. Additionally, the stability of polyplexes in the presence of serum proteins is found to be excellent. Finally, the multi-responsive hyperbranched copolymers were evaluated regarding their cytotoxicity via in vitro experiments on HEK 293 non-cancerous cell lines and found to be sufficiently non-toxic. Based on our results, these polyplexes could be useful candidates for gene delivery and related biomedical applications.

## 1. Introduction

In recent years, gene therapy has been one of the greatest challenges in the treatment of several genetic and inherited diseases via targeted biomaterial-based nanomedicines delivered to contaminated cells and tissues [1]. One of the challenging barriers to gene therapy is the optimization design of suitable biomedical vectors. Specifically, the design and development of an ideal gene carrier will have to satisfy certain conditions. An effective gene carrier should be capable of condensing the large molecules of deoxyribonucleic acid (DNA) to appropriate dimensions for cellular uptake and, at the same time, protect them from extra- and intracellular nuclease degradation. Additionally, a suitable gene carrier must be nonviral with low toxicity, lack of immunogenicity, and must generally be an economically viable product [2,3,4,5].

Nowadays, the polymer community has been focused on the synthesis of polymeric biomaterials in order to treat such diseases. Nonviral vectors based on cationic polymers [6,7,8,9,10] and lipids [11,12,13,14] have been proven to be efficient carriers in gene therapy strategies. Nevertheless, the utilization of cationic copolymers as gene-delivery vehicles has some remarkable advantages in comparison with the use of lipids. In particular, the synthesis and design of polymer-DNA complexes can be controlled, and polyplexes of relatively small size and narrow size distribution can be produced [15,16], showing high stability against nuclease degradation and potential augmentation of the payload of the vectors. 

Cationic polymers could be synthesized using a versatile chemical reaction with a controlled molecular weight, chemical composition, and macromolecular architecture [17,18,19]. These parameters are very important for designing and developing effective gene-delivery polymeric systems. Cationic polymers, due to their high positively charged amine groups, are capable of forming polyplexes after simple mixing with DNA molecules. Electrostatic interactions take place between the positively charged amine groups of the polymers and the negatively charged phosphate groups of the DNA [20,21]. 

Hyperbranched copolymers (HBC) have gained interest among polymer scientists because of their unique properties [22,23,24,25]. Additionally, hyperbranched copolymers have received great recognition for their utilization as biomaterials in biomedical applications [26,27,28]. These polymers exhibit different properties and structural characteristics in relation to their linear analogs due to their different macromolecular architecture [29,30]. In particular, these polymers are distinguished by their high degree of polymerization, having a large number of internal/external active groups. Hyperbranched copolymers have the advantage of being synthesized by one-step polymerization compared to the multi-stage synthesis of dendrimers. Therefore, these polymers are ideal materials for large-scale industrial applications [31,32]. In addition, some interesting characteristics of hyperbranched copolymers are their low-level viscosity as well as their compact conformations because of their hyperbranched structure. These characteristics make hyperbranched copolymers a potential platform for a wide range of biomedical applications, such as drug [33,34,35] and gene delivery [36,37,38], as well as tissue engineering [39,40,41]. 

Important classes of polymeric materials for gene delivery are the so-called smart polymers. Smart polymers are also well-known as stimuli-responsive polymers, able to respond to environmental changes, and have been used in the field of biological science, nanomedicine, and tissue engineering [42,43]. Temperature and pH stimuli, as well as the alternations in the ionic strength, are the most common stimuli, especially in biological environments. A plethora of research articles has reported on thermo-responsive polymers, which are distinguished by a low critical solution temperature (LCST) [44]. The polymeric system below LCST is completely soluble in water, while above LCST, the polymer is partially soluble, resulting in reduced polymer-water interactions and the aggregation of polymer chains. The pH-sensitive polymers contain monomers with ionizable groups and display alternations in their conformation due to pH changes. Cationic polymers are also well-known as polyelectrolytes, consisting mainly of amino groups. Poly(2-[dimethylamino] ethyl methacrylate) (PDMAEMA) and poly(2-[diisopropylamino] ethyl methacrylate) (PDIPAEMA) polymers [45,46] contain amino groups where their protonation converts them into highly hydrophilic polymers below the respective pK_a_ values. 

Herein, we report on the ability of multi-responsive P(OEGMA-co-DIPAEMA) hyperbranched polyelectrolyte copolymers to interact with small DNA molecules. For this reason, three hyperbranched copolymers were synthesized in our previous work [47] with different ratios of hydrophobic to hydrophilic components. These hyperbranched copolymers consisted of a thermo- and pH-responsive polymer, PDIPAEMA (T_cp_ ca. 27–60 °C) [48,49], which, below its pKa (6.2) [50], is converted to cationic polyelectrolyte and a hydrophilic polymer, POEGMA, which provides stealth properties to the polymer nanostructures. The formed polyplexes were studied in detail via several physicochemical characterization methods. The changes in the mass and the size of polyplexes were determined by Dynamic light scattering (DLS) measurements, where the DNA binding capability of the hyperbranched polyelectrolytes was evaluated by Electrophoretic light scattering (ELS) and Fluorescence spectroscopy (FS) measurements. Furthermore, the colloidal stability of these polyplexes was investigated under physiological conditions. Last, an in vitro cytotoxicity study was undertaken to assess the biocompatibility of these cationic hyperbranched polyelectrolyte copolymers.

## 2. Materials and Methods

### 2.1. Materials

Deoxyribonucleic acid sodium salt (DNA, with ~113 bp) from salmon testes and ethidium bromide (EtBr), used as the fluorescent DNA intercalating agent for the complexation investigation, were received from Sigma-Aldrich. Phosphate-buffered saline tablets (PBS, 98%) and fetal bovine serum (FBS) were received from Sigma-Alrdrich, Athens, Greece).

### 2.2. Hyperbranched P(OEGMA-co-DIPAEMA) Polyelectrolyte Copolymers Synthesis

**Scheme 1:**
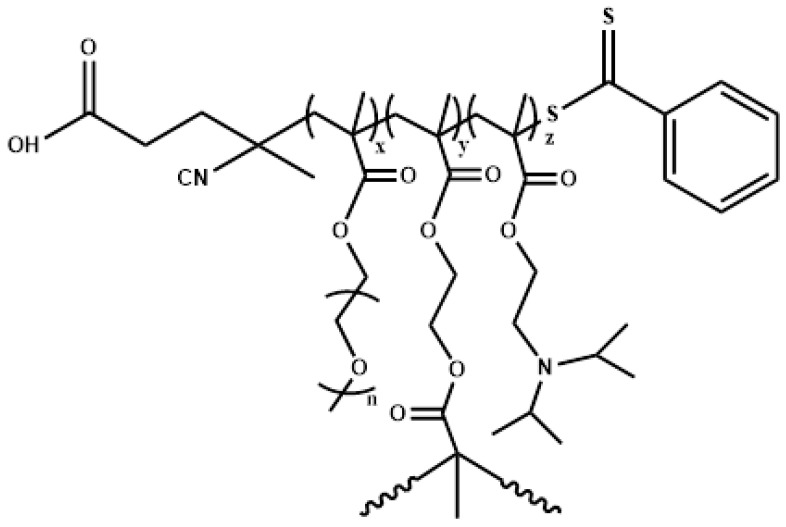
Chemical structure of the P(DIPAEMA-co-OEGMA) hyperbranched copolymers.

Reversible Addition Fragmentation Chain Transfer (RAFT) polymerization was used to synthesize three hyperbranched copolymers via one-step polymerization, and the ethylene glycol dimethacrylate (EGDMA) monomer was utilized as the branching agent. The chain transfer agent was the 4-cyano-4(phenyl-carbonothioylthio) pentanoic acid (CPAD), and 2,2′-azobis (isobutyronitrile), (AIBN) was used as the radical initiator. Scheme 1 represents the chemical structure of P(OEGMA-co-DIPAEMA) hyperbranched copolymers. More details about the synthesis of these copolymers are mentioned in our previous work [47]. Table 1 summarizes the molecular characteristics of the hyperbranched copolymers utilized in this study.

It is worth noting that the molecular weights calculated from the SEC method are apparent ones. Additionally, by using static light scattering (SLS) in tetrahydrofuran (THF), it was possible to determine the apparent weight average molecular for the hyperbranched copolymers. Calibration of our SEC instrument was performed using linear polystyrene standards, which is why the molecular weight (mass) from the SEC method is much lower than that of the SLS method. The discrepancy between the molecular weights determined by SEC (a technique based on hydrodynamic volume) and those determined by SLS (an absolute technique able to determine the molar mass of the copolymer) is substantial proof that the obtained copolymers have a hyperbranched molecular structure.

### 2.3. Preparation of P(OEGMA-co-DIPAEMA)/DNA Polyplexes in Aqueous Media

The hyperbranched copolymer/DNA polyplexes were prepared by simple mixing of the polyelectrolyte and DNA stock solutions in the desired amine/phosphate ratios. NaCl (0.01 M) aqueous solution was used to prepare all stock solutions by direct dissolution of the copolymers and DNA. The mixed solutions were achieved at desired N/P ratios in the 0.25–4 N/P range by adding the proper quantity of DNA solution at room temperature into the polyelectrolyte solution under gentle stirring. A typical example of the preparation of hyperbranched P(OEGMA-co-DIPAEMA)/DNA polyplexes at acidic conditions (pH = 3) is mentioned below: Initially, 4 mg of the copolymer was directly dissolved in 8 mL of 0.01 M NaCl solution (C = 5 × 10^−4^ g/mL) and left at room temperature for complete dissolution. Afterward, 7 mg of DNA was directly dissolved in 35 mL of 0.01 M NaCl solution (C = 2 × 10^−4^ g/mL). The last step was the addition of an appropriate volume of DNA stock solution into the copolymer solution in order to have the desired N/P ratio. For all cases, the same process was followed. Solution pH was varied by adding appropriate volumes of 1 M HCl and NaOH aqueous solutions. 

### 2.4. FBS Interactions with P(OEGMA-co-DIPAEMA)/DNA Polyplexes

The solutions of P(OEGMA-co-DIPAEMA)/DNA polyplexes with clarified FBS were performed in filtered PBS, utilizing 50 μL of each polyplexes solution and different FBS/PBS ratios. Regarding this protocol, 50 μL of each polyplexes solution was mixed with 3 mL FBS/PBS (1/9 *v*/*v*) ratio. The mixed solutions were left for 1 h to equilibrate before measurements.

### 2.5. MTT Assay

HEK293 non-cancerous cell lines derived from the human embryonic kidney (commercially available from the ATCC 293 [HEK-293] CRL-1573™) were grown at a standard temperature of 37 °C, utilizing a DMEM high-glucose culture solution comprising 10% FBS, 2 mmol/L glutamine, 100 IU/mL penicillin, and 100 mg/mL streptomycin. Approximately every 48 h, the nutrient medium was replaced, and cells were transferred on a weekly basis, utilizing typical trypsin-EDTA concentrations. After reaching adequate accumulation, the cells were removed to a 96-well plate, and 5000 cells/well were seeded. A steri-cycle CO_2_ incubator was utilized for the incubation (HEPA Class 100, Thermo Electron Corporation^®^, Waltham, MA, USA). Utilizing a microscope enabled the assessment of the HEK293 cell viability. Optical evaluation was also accomplished by possible discoloration of the 96-well plate (transition toward a more yellowish color indicated cell stress or possible culture contamination). The incubation time was regulated at 24 h at 37 °C. The range of copolymer concentrations was between 25–500 μg/mL. All the processes were performed in a sterile environment. All experiments were replicated twice (*n* = 2), while the data analysis was performed less than a week from the initial preparation of the polymeric system.

### 2.6. Methods

#### 2.6.1. Dynamic Light Scattering

DLS measurements were implemented, utilizing a wide-angle light scattering photometer by ALV GmbH, CGS-3. This instrument consisted of a He-Ne 22 mW laser source, a compact goniometer system with an Avalanche photodiode detector interfaced with an ALV/LSE-5003 electronics unit, and an ALV-5000/EPP multi-tau digital photon correlator. The instrument is connected to a Polyscience model 9102 bath circulator for controlling the temperature of the measuring cell. The Contin algorithm was used to analyze the DLS data. The size, polydispersity index, and scattering intensity of the formed nanostructures were recorded using DLS instrument software. The parameters obtained by the techniques implemented have some typical errors (indicatively for DLS: I (intensity) < 2% error, R_h_ < 5% error based on five repeated measurements). The size data and graphs illustrated below correspond to measurements at a 90° angle. All solutions were filtered with a 0.45 μm hydrophilic PVDF filter prior to measurements. 

All the prepared polyplexes solutions were left to equilibrate overnight before measurements.

#### 2.6.2. Electrophoretic Light Scattering

A Nano Zeta Sizer (Malvern Instruments Ltd., Malvern, UK) instrument equipped with a 4 mW He-Ne laser, operating at 633 nm, and measuring at a scattering angle of 173°, was used for ELS experiments. Surface charge (ζ-potential) values reported were the average of approximately 15 repeated measurements analyzed by the Smoluchowski equation (<5% error on the values reported for the ζ-potential).

#### 2.6.3. Fluorescence Spectroscopy

The capability of the P(OEGMA-co-DIPAEMA) hyperbranched polyelectrolytes to form polyplexes with small DNA molecules was achieved by ethidium bromide (EtBr) quenching assay through fluorescence spectroscopy technique. Firstly, a DNA stock media (1 × 10^−4^ g/mL) was prepared, followed by the addition of EtBr ([EtBr] = [P]/4). Afterward, the DNA medium was titrated utilizing a proper volume of polymer stock solution in the range of N/P ratio from 0.0 to 4.0. For each addition of the polymer, the system was left for 15 min to equilibrate before measurement. Fluorescence spectroscopy experiments were recorded utilizing a Fluorolog-3 Jobin Yvon-Spex spectrofluorometer (modelGL3- 21), and the excitation and emission wavelengths for the measurements were at 535 nm and 600 nm, respectively.

## 3. Results and Discussion

### 3.1. Complexation Behaviour of Hyperbranched Polyelectrolyte Copolymers with DNA in Aqueous Solutions

**Figure 1:**
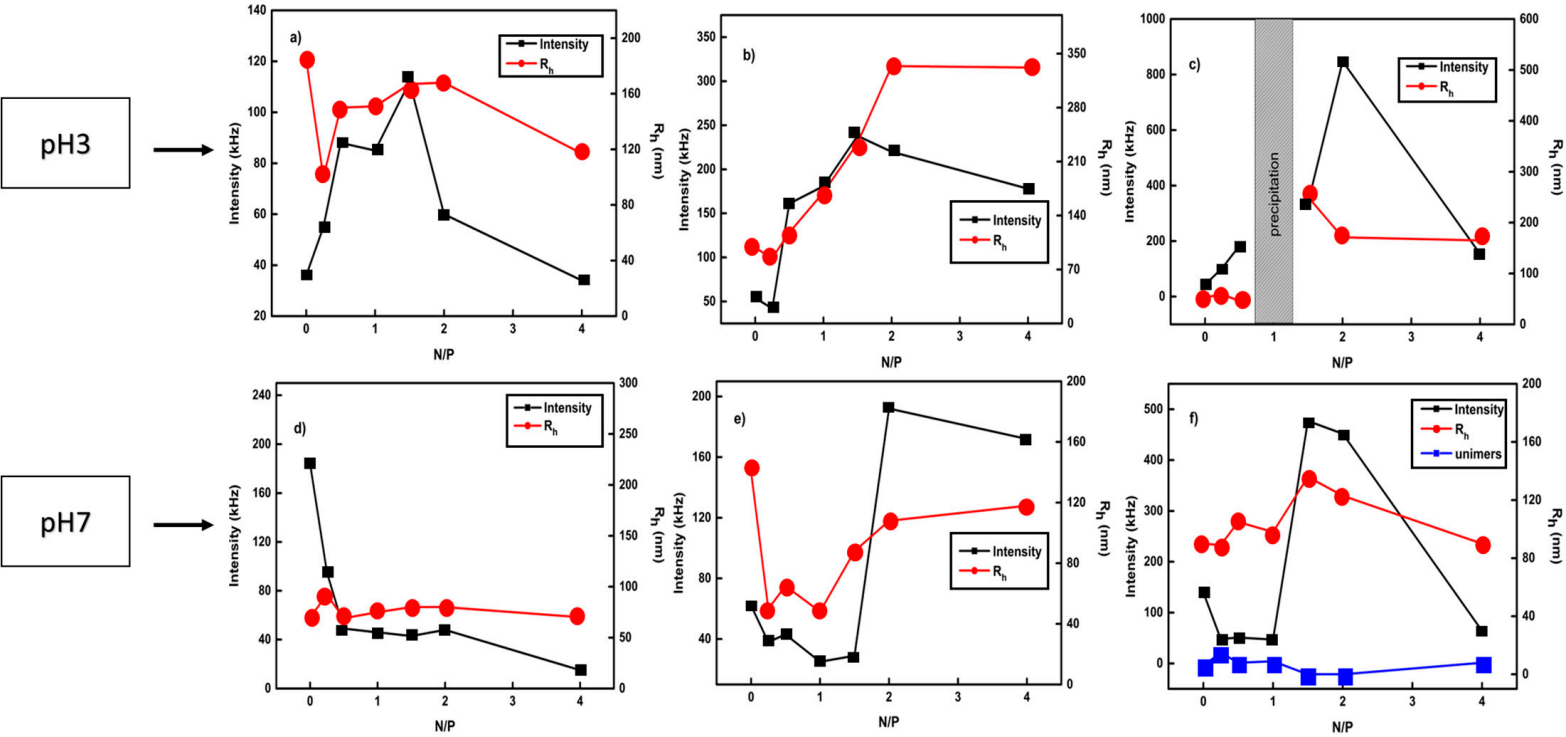
Variation of scattered intensity and R_h_ as a function of pH and N/P from light scattering experiments for the P(OEGMA-co-DIPAEMA)/DNA polyplexes. (**a**) HBC-1 polyplexes at pH 3, (**b**) HBC-2 polyplexes at pH 3, (**c**) HBC-3 polyplexes at pH 3, (**d**) HBC-1 polyplexes at pH 7, (**e**) HBC-2 polyplexes at pH 7 and (**f**) HBC-3 polyplexes at pH 7. Blue line (refers to R_h_ y axis) corresponds to the unimer nanoparticles, which are single polymeric chains. Five repeated measurements were performed for each point. Size of the points in the diagrams refers to error bars in the y-axes. C_copolymer_ = 5 × 10^−4^ g/mL.

The self-assembly of P(OEGMA-co-DIPAEMA) hyperbranched copolymers has been studied extensively in our previous work [47]. The complexation behavior of the P(OEGMA-co-DIPAEMA) hyperbranched polyelectrolyte copolymers with small DNA molecules through electrostatic interactions between the positively amine groups and negative phosphate groups was investigated extensively by DLS measurements. In particular, the DLS technique was used to identify the hydrodynamic radii (R_h_) of the polyplexes, the alterations of the scattered light intensity (which is correlated with the mass of the polyplexes), and the size polydispersity index of them. The formed P(OEGMA-co-DIPAEMA)/DNA polyplexes were studied at various N/P ratios in the 0.25–4 range. The complexation character was explored at two different pH values (pH 3 and pH 7) due to the pH responsiveness of the PDIPAEMA component. It is worth mentioning that the PDIPAEMA polymeric chains at acidic conditions are fully protonated, while in neutral environments are partially protonated. The DLS data for all polyplexes are presented in Figure 1. From DLS results at pH3, a gradual increment in scattered intensity is observed for all polyplexes as the N/P ratio increases from 0.25 to 1.5 and then reduces until the N/P ratio is 4. As mentioned above, at acidic conditions, the amine groups of the PDIPAEMA component are fully protonated; thus, the formed complexes appear to be well-organized. It seems that all the protonated amine groups are available to interact with the negative phosphate groups. By increasing the N/P ratio from 2 to 4, a reduction in mass is observed. This is probably due to the reduced number of negative phosphate groups to electrostatically interact with the positive amino groups present in the solution, resulting in the formation of complexes of lower mass. As regards the value of the hydrodynamic radius in the case of HBC-1, no significant changes in the size of polyplexes are observed. The opposite appears to be true in the case of HBC-2 and HBC-3 polyplexes. Specifically, an increment in the size of them is observed by increasing the N/P ratio. The presence of higher content of fully protonated DIPAEMA segments creates the tendency for rather extended macromolecular conformations. It is also worth mentioning that in the HBC-3 system, with the higher content of hydrophobic PDIPAEMA component, the formed complexes in the N/P = 1 ratio are not stable and precipitate from the solution. This is due to complete neutralization of the positive charges of the cationic units, which are accessible by the negative charges of the phosphate groups of the DNA, resulting in the precipitation of the formed nanoparticles. 

At pH 7, the amino groups of PDIPAEMA are partially protonated; thus, the polymeric system switches to a more hydrophobic state. In the case of HBC-1 at pH 7, an initial rather steep reduction of scattered intensity from N/P = 0.25 to 0.5 is observed, and then a plateau is attained, showing no particular changes in the mass of the nanoparticulate complexes. From this fact, we may infer that the system has a tendency to form less well-organized complexes. This may be due to three reasons. The first one is that in the current situation, the system has shifted to a more hydrophobic state, and therefore the amino groups are partially protonated, unable to strongly interact with negative phosphate groups. The second reason is that the HBC-1 copolymer has a low content of PDIPAEMA (thus, the number of available amine groups for complexation is rather low). The third reason is that due to the dense branched structure of the copolymer as a result of its macromolecular architecture, the DNA molecules cannot bind with all available protonated amino groups. In the case of the HBC-3 copolymer, a sharp increase in scattered intensity is observed in the region from N/P = 1 to 1.5, which is followed by a reduction until the N/P ratio is 4. An increase in the scattered intensity involves an increase in the mass of polyplexes because, apparently, there are more positive amino groups interacting with the negative phosphate groups, and therefore better complexation between DNA and polymer is achieved. Furthermore, concerning the size of the complexes, the formation of two distinct size populations is observed over the whole range of the N/P ratio. It seems that the increase in the hydrophobic element leads to the splitting of the complexes into smaller ones with the parallel existence of large ones. An interesting observation can be made comparing the behavior of HBC-1 and HBC-3 copolymers. It appears that the value of the hydrodynamic radius of the nanoparticles of the HBC-1 is between the values of the hydrodynamic radius of the two populations of the HBC-3. Furthermore, HBC-2, which is in an intermediate situation with respect to the chemical composition, does not show two populations of complexes. Our hypothesis is that when there is a large amount of PDIPAEMA in the copolymer, the complexes cannot maintain their original structure in the solution and split into complexes of different sizes. Also, comparative size distributions from DLS for P(OEGMA-co-DIPAEMA)/DNA polyplexes at N/P 2 are presented in Supplementary Material (Figure S1).

**Figure 2:**
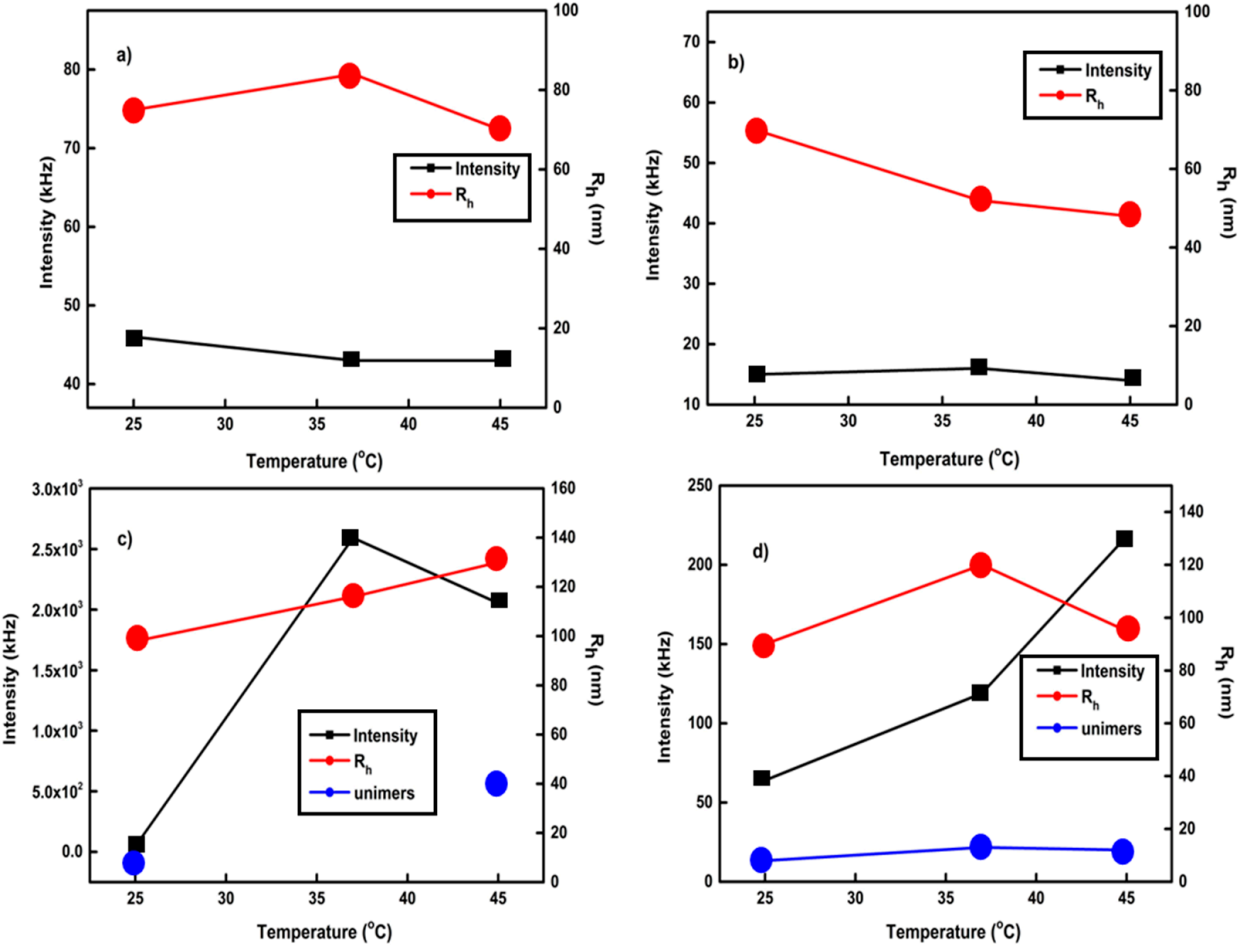
Variation of scattered intensity and R_h_ as a function of temperature from light scattering experiments for the P(OEGMA-co-DIPAEMA)/DNA polyplexes at pH 7. (**a**) HBC-1 polyplexes at N/P 1, (**b**) HBC-1 polyplexes at N/P 4, (**c**) HBC-3 polyplexes at N/P 1 and (**d**) HBC-3 polyplexes at N/P 4. Five repeated measurements were performed for each point. Size of the points in the diagrams refers to error bars in the y-axes. C_copolymer_= 5 × 10^−4^ g/mL.

A subsequent study was focused on investigating the alternations in the size and mass of the complexes by increasing solution temperature. All measurements were accomplished at pH 7. Comparative graphs for HBC-1 and HBC-3 hyperbranched copolymers are illustrated in Figure 2. From DLS measurements, it is almost clear that the most strongly affected system is HBC-3. HBC-3 hyperbranched copolymer has a higher content of PDIPAEMA component, thus, may show a stronger effect on the size and mass of the formed polyplexes by increasing temperature. It is also observed between the polymeric systems investigated that the scattered intensity is clearly higher in the copolymer where the hydrophobic component predominates. Furthermore, an interesting observation is that, in the case of the HBC-3 copolymer at the N/P 1 ratio, the mass of nanoparticles seems to be much higher in contrast to the N/P 4 ratio. Presumably, this is happening due to the balance that exists between the positively charged groups and the negatively charged groups; thus, most of the available amino groups interact with the phosphate groups of DNA, leading to the formation of well-defined and compact polyplexes. The hydrophobic subdomains formed by DIPAEMA segments may contribute to this behavior.

**Figure 3:**
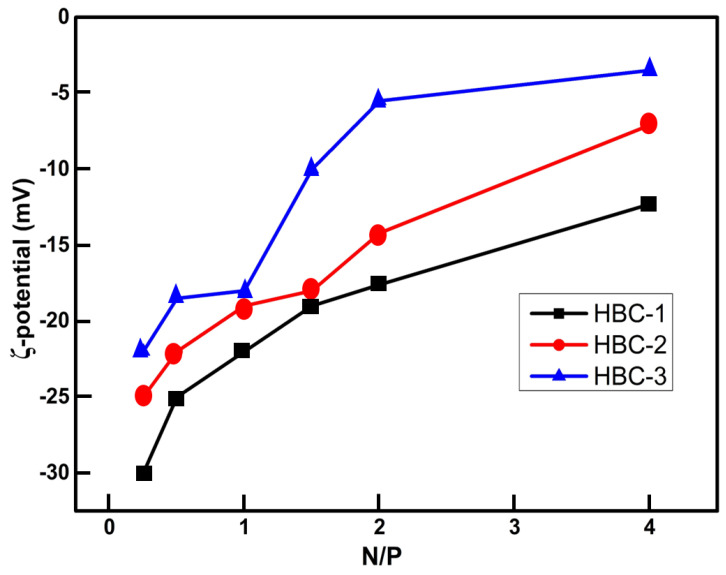
Electrophoretic light scattering results of P(OEGMA-co-DIPAEMA) polyplexes at pH7. Three repeated measurements were implemented for each point. Size of the points in the diagrams refers to error bars on the y-axis. C_copolymer_= 5 × 10^−4^ g/mL.

Electrophoretic light scattering measurements were recorded in order to investigate the surface charge of the P(POEGMA-co-DIPAEMA) hyperbranched polyplexes. The acquired data were accomplished at pH7 and 25 °C. ELS results presented in Figure 3 reveal negative values for all hyperbranched polyplexes at all N/P ratios. It is speculated that this is happening because of the macromolecular architecture of the copolymers. In particular, due to dense branching, the DNA molecules are localized on the periphery of the hyperbranched structure of the copolymer. In addition, the large negative ζ-potential values could be interpreted as a strong complexation of polymers with DNA, especially when the polymer is not predominant in the mixture. Moreover, it is evident that in all polymeric systems, when there is a higher PDIPAEMA content, the ζ-potential for the complexes shifts to less negative values. This is due to the higher number of protonated amino groups originating from the PDIPAEMA component.

### 3.2. Effect of Solution Ionic Strength on the Formed Polyplexes

**Figure 4:**
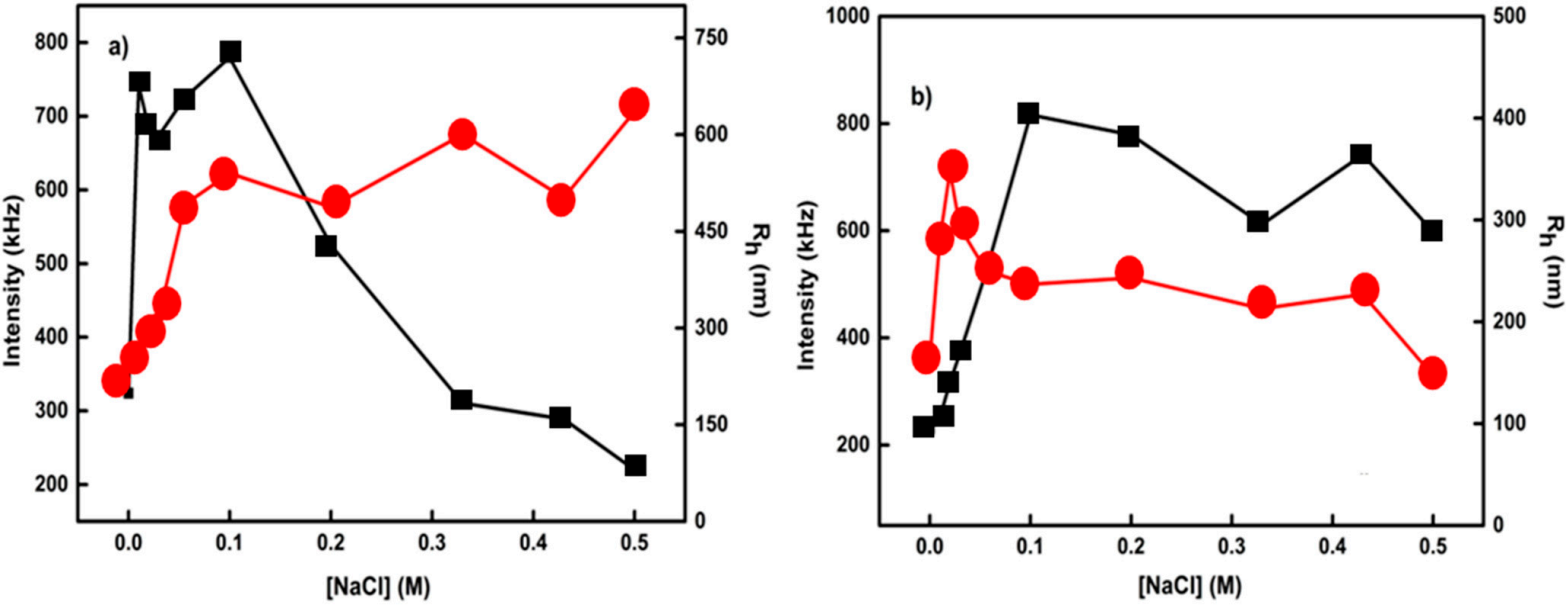
Light scattering results as a function of ionic strength for HBC-3 polyplexes at pH 3 (black data: intensity, red data: R_h_). (**a**) HBC-3 polyplexes at N/P 1.5 and (**b**) HBC-3 polyplexes at N/P 4. Five repeated measurements were implemented for each point. Size of the points in the diagrams refers to error bars on the y-axes. C_copolymer_ = 5 × 10^−4^ g/mL.

The next step was to examine the behavior of these hyperbranched polyelectrolyte carriers in acidic conditions. Utilizing DLS, it was possible to evaluate the ability of the formed polyplexes to change their structural conformation as a response to salt concentration changes. Figure 4 illustrates the alteration of the size and mass through titration (addition) of NaCl into the solutions of polyelectrolyte/DNA complexes. From the DLS measurements presented in Figure 3, it is evident that in the case with a relative balance between the amino groups of DIPAEMA segments in the copolymer and the phosphate groups of DNA molecules, a gradual increase in the size of polyplexes is observed. The increase in the hydrodynamic radius reveals the swelling of the polyplexes due to the increment of electrostatic interactions among the components. As far as the scattered intensity is concerned, there is an increase in the first addition. By increasing the salt concentration, a plateau is reached, and thereafter, a sharp reduction in the mass of the polyplexes is revealed, demonstrating the decomposition of the initially formed polyplexes. The opposite happens when there is a higher PDIPAEMA content. In particular, a sharp increase in the mass of polyplexes is observed until 0.1 M NaCl, and thereafter a plateau exists until 0.5 M NaCl is present. It seems that, in this N/P ratio, there is a stronger electrostatic interaction between the hyperbranched polyelectrolyte and DNA molecules. Regarding the hydrodynamic radius, no significant changes are presented as a function of salt concentration except for the two initial salt additions.

### 3.3. Temporal Stability Studies of the Polyplexes 

**Figure 5:**
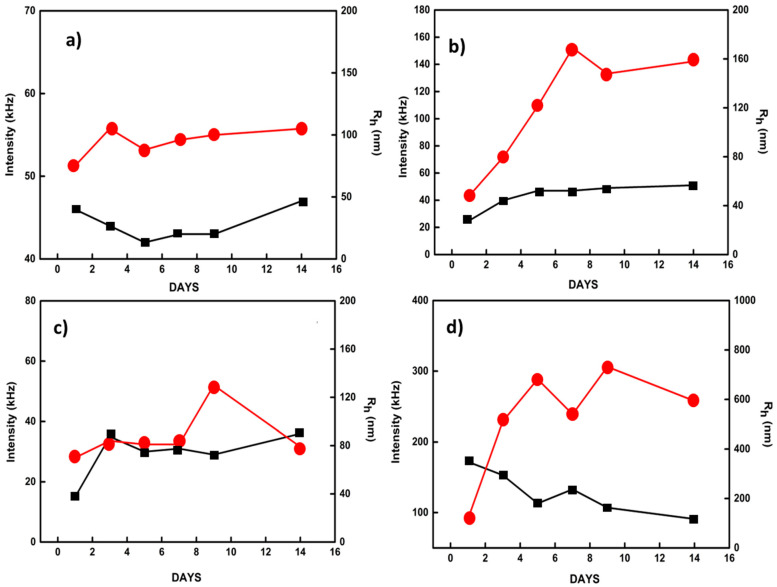
DLS measurements of P(OEGMA-co-DIPAEMA)/DNA polyplexes with respect to the time at pH 7 (black data: intensity, red data: R_h_). (**a**) HBC-1 polyplexes at N/P 1, (**b**) HBC-2 polyplexes at N/P 1, (**c**) HBC-1 polyplexes at N/P 4 and (**d**) HBC-2 polyplexes at N/P 4. Five repeated measurements were implemented for each point. Size of the points in the diagrams refers to error bars on the y-axes. C_copolymer_ = 5 × 10^−4^ g/mL.

After the physicochemical investigation of the hyperbranched polyplexes, the next task was to evaluate the temporal stability of the formed polyplexes. Figure 5 presents characteristic plots from DLS results. DLS was used to determine the changes in the size and mass of the polyplexes at pH 7. In the case of HBC-1 polyplexes in both N/P ratios, no significant changes in the mass and size of the nanoparticles are observed. It appears that the formed polyplexes retain their original sizes, which demonstrates the relatively stable complexation between the polymer and the DNA molecules. The stability of the particular polyplexes should also be attributed to the stronger hydrophilic character of HBC-1 copolymer, having the highest OEGMA content. In the case of HBC-2, where the PDIPAEMA content is increased, remarkable changes in the size of the nanoparticles are observed. Specifically, at the N/P 4 ratio, where the concentration of the polymer is substantially increased, a relative decrease in the mass of the nanoparticles is observed, which is accompanied by a simultaneous increase in the hydrodynamic radius. This effect shows the gradual decomposition of the polyplexes over time, most probably due to the fact that originally not all available amino groups could bind to the DNA phosphate groups. Generally, by increasing the hydrophobic element in the polymeric system, the polyplexes are not stable with respect to the time passed after their preparation.

### 3.4. Ethidium Bromide Quenching Assay on Polyplexes

**Figure 6:**
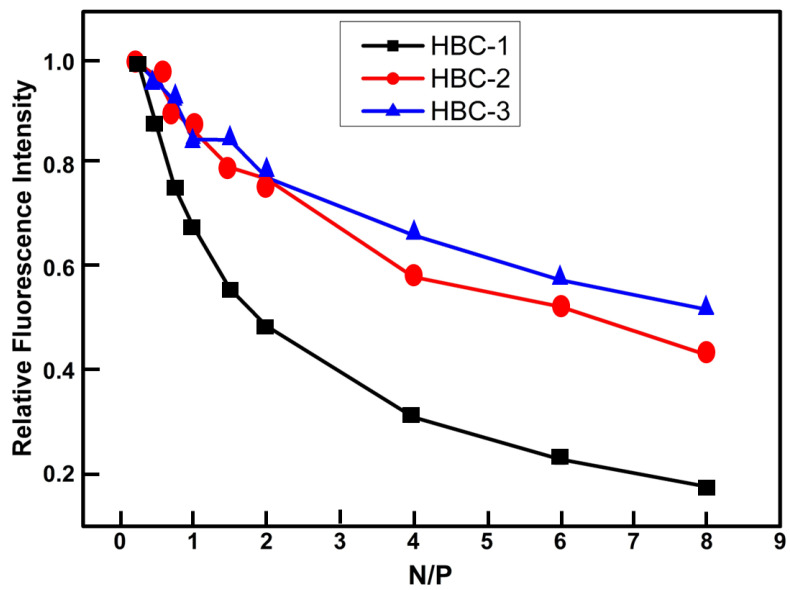
Ethidium bromide fluorescence quenching of P(OEGMA-co-DIPAEMA) polyplexes at pH 7. Size of the points in the diagrams refers to error bars on the y-axis. Measurements were repeated three times. C_copolymer_ = 1 × 10^−3^ g/mL.

Ethidium bromide (EtBr) is a fluorescent dye molecule interacting with DNA chains by intercalating between its base pairs, exhibiting strong fluorescence [51,52]. Subsequently, during the complexation of DNA with the cationic copolymer, the intercalated EtBr is displaced from the DNA double helix, and the fluorescent intensity reduces, demonstrating in this way the ability of the cationic polymer to form stable polyplexes with DNA. Therefore, fluorescent spectroscopy measurements were carried out to identify the strength of copolymer/DNA interaction through the relative fluorescence intensity of EtBr in the formed polyplexes as a function of the N/P ratio. Figure 6 represents characteristic curves of relative fluorescent intensity vs. the N/P ratio at pH 7. In all cases, a gradual reduction in fluorescent intensity is observed. It is worth mentioning that in all cases, the intercalated EtBr is not completely displaced from the double helix of the DNA. This is probably happening because of the dense conformation of the hyperbranched copolymer; thus, the DNA cannot bind to all available protonated amine groups of the DIPAEMA chain segments. However, in the case of HBC-1 copolymer with the lowest content of amino groups, the greatest quenching of the fluorescent intensity of EtBr occurs. It seems that there is a greater interaction between the negatively charged DNA molecules with the positively charged amino groups of DIPAEMA segments due to fewer spatial constraints originating from the copolymer conformation in this case.

### 3.5. FBS Interactions with Polyplexes

**Figure 7:**
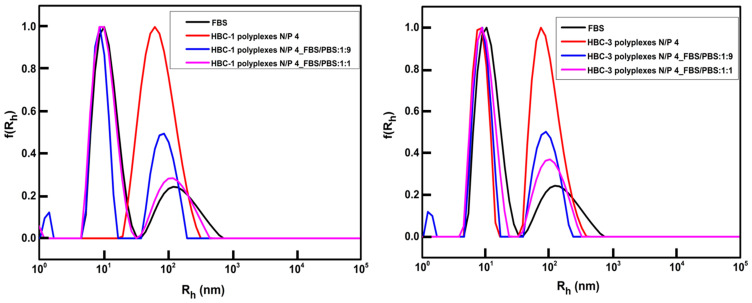
Size distribution graphs of P(OEGMA-co-DIPAEMA)/DNA polyplexes under physiological conditions. Each curve is an average of five repeated measurements. C_copolymer_ = 3 × 10^−3^ g/mL.

After studying the stability of the formed polyplexes with respect to time, it was necessary to study the P(OEGMA-co-DIPAEMA)/DNA polyplexes under simulated physiological conditions in order to determine possible interactions with blood proteins. For this reason, DLS stability measurements were conducted for hyperbranched polyplexes in FBS media in order to specify possible changes in their physicochemical properties. DLS results were obtained at 25 °C and at 90°angle after 1 h of mixing polyplexes into the FBS/PBS (1:9 and 1:1 *v*/*v*) mixed medium. Figure 7 represents comparative graphs of size distributions of P(OEGMA-co-DIPAEMA)/DNA polyplexes before and after mixing with FBS/PBS solution. Based on the DLS results illustrated in Figure 7, it seems that no significant interaction of the existing polyplexes with the FBS/PBS medium is taking place. In particular, the creation of a new peak, indicating further aggregation of the formed polyplexes, is not observed. Therefore, polyplexes of hyperbranched cationic copolymers reveal remarkable stability in a simulated biological medium.

### 3.6. In Vitro Cytotoxicity Studies

**Figure 8:**
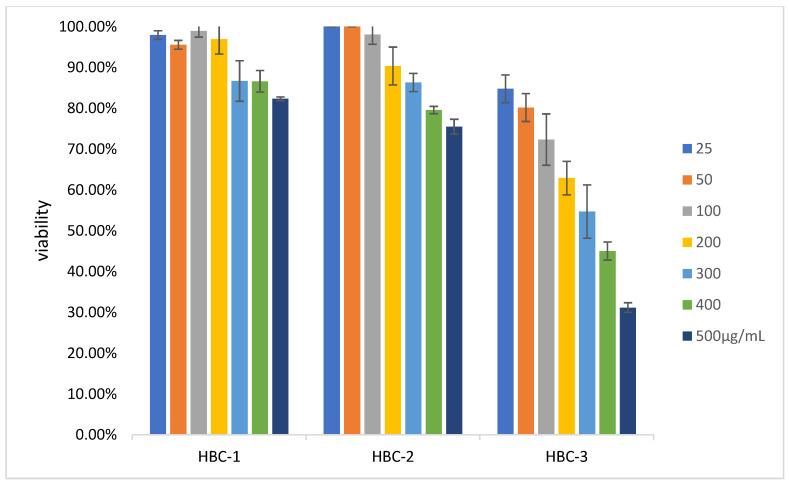
Cell viability vs. different polymer concentrations of P(OEGMA-co-DIPAEMA) hyperbranched copolymers. Bars denote standard deviation of three repeats.

All P(OEGMA-co-DIPAEMA) hyperbranched polyelectrolyte copolymers were investigated for their biocompatibility using an in vitro cytotoxicity experiment. Figure 8 represents the non-cancerous HEK293 cell viability between the different hyperbranched copolymer concentrations. All polymeric systems exhibit dose-dependent cytotoxicity. According to the cytotoxicity results, it is evident that increasing the PDIPAEMA component leads to a significant reduction in cellular viability. In the case of HBC-3 hyperbranched copolymer, even at the lowest polymer concentration (25μg/mL), the viability of the cells did not exceed 85%. A completely different pattern is observed in the case of HBC-1 copolymer, where the cell viability is over 80% for all the examined concentrations. A similar trend prevails in the case of HBC-2 copolymer, where up to 100μg/mL polymer concentration, the cellular viability is almost 100%. As for the HBC-1 copolymer, when its concentration is 200 μg/mL, the viability of cells is close to 100%. Furthermore, the HBC-1 and HBC-2 are sufficient biocompatible polymeric systems; the cell viability is over 75% in both cases. 

Generally, the polymeric nanoparticles containing the highest content of PDIPAEMA are more cytotoxic, except for low polymer concentrations (i.e., 25, 50, 100μg/mL). The hydrophobic-to-hydrophilic ratio seems to play a major role in the biocompatibility of the copolymers. We speculate that this is attributed to different interactions, inducing changes in self-organization and shape of nanostructures apart from positive charge effects.

## 4. Conclusions

In this paper, the multi-responsive P(OEGMA-co-DIPAEMA) cationic hyperbranched polyelectrolyte copolymers, possessing different hydrophobic-to-hydrophilic ratios, were capable of forming polyplexes with short DNA molecules in aqueous media through electrostatic interactions. The complexation behavior was extensively studied using light scattering techniques. Utilizing fluorescence spectroscopy, we confirmed the binding of protonated amino groups of DIPAEMA segments with negative phosphate groups of DNA macromolecules. Specifically, the polyplexes containing higher PDIPAEMA content revealed a low level of fluorescence intensity quenching, presumably due to the dense branching of the copolymers. A dynamic light scattering technique was utilized to evaluate the physicochemical characteristics of the polyplexes in aqueous media as a function of pH and temperature. DLS measurements revealed that the mass and size of the formed polyplexes show a strong dependence on variations in the chemical composition of the hyperbranched copolymers, the N/P ratio, the solution pH, and temperature, as well as the ionic strength. Temporal stability studies of formed polyplexes revealed their stability with respect to time in certain cases. Additionally, the P(OEGMA-co-DIPAEMA)/DNA nanoparticles presented remarkable stability under simulated physiological conditions. Furthermore, in vitro cytotoxicity studies evidenced significant biocompatibility, especially for the copolymers with a lower content of PDIPAEMA. In conclusion, the findings of this research show the potential of these copolymers to be utilized as gene-delivery vehicles.

## Figures and Tables

**Table 1:** Molecular characteristics of P(OEGMA-co-DIPAEMA) hyperbranched copolymers.

Sample	M_w_ ^a^ (g/mol) (×10^4^)	M_w_ ^b^ (g/mol) (×10^4^)	M_w_/M_n_ ^b^	%wt PDIPAEMA ^c^	%wt POEGMA ^c^
HBC-1	33.3	1.2	1.21	10	90
HBC-2	48.6	0.8	1.19	29	71
HBC-3	35.1	1.1	1.24	54	46

^a^ Determined by static light scattering (SLS). ^b^ Determined by size exclusion chromatography (SEC). ^c^ Determined by nuclear magnetic resonance (^1^H-NMR).

## Data Availability

The data presented in this study are available on request from the corresponding author.

## Supplementary Materials

The following supporting information can be downloaded at: https://www.mdpi.com/article/10.3390/pharmaceutics15061627/s1, Figure S1: Comparative size distributions from DLS for P(OEGMA-co-DIPAEMA)/DNA polyplexes at N/P 2.
