# Supplementary material for: Novel Multi-Responsive Hyperbranched Polyelectrolyte Polyplexes as Potential Gene Delivery Vectors
Source: Pharmaceutics. 2023 May 30;15(6):1627. doi: 10.3390/pharmaceutics15061627 (PMC10301639; doi:10.3390/pharmaceutics15061627)
Supplement: Supplementary file 1 [file pharmaceutics-15-01627-s001.zip › pharmaceutics-2386181-supplementary.pdf]

Supplementary Materials

Novel Multi-Responsive Hyperbranched Polyelectrolyte Polyplexes as Potential Gene Delivery Vectors

Dimitrios Selianitis [1], Hector Katifelis [2], Maria Gazouli [2] and Stergios Pispas [1,*]

Dynamic Light Scattering measurements for the P(OEGMA-co-DIPAEMA) polyplexes

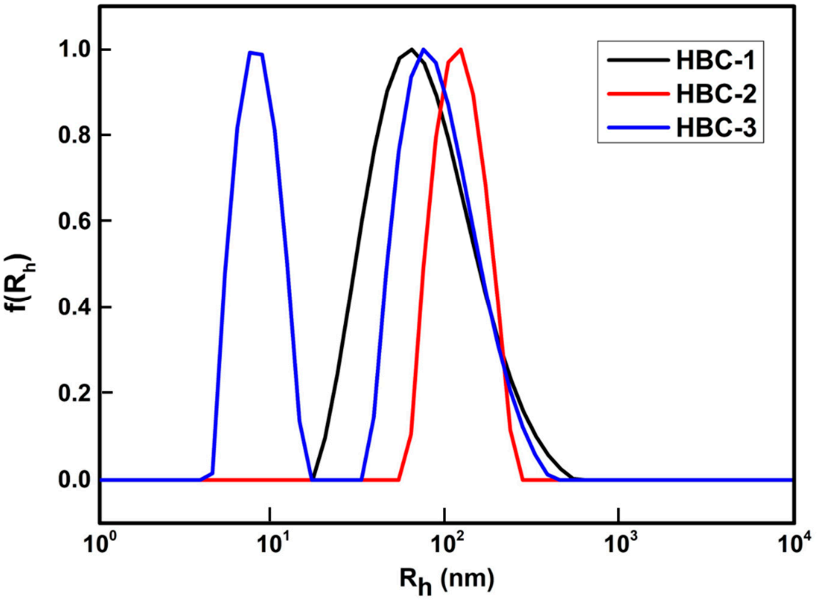

Figure S1: Comparative size distributions from DLS for P(OEGMA-co-DIPAEMA)/DNA polyplexes at N/P 2.
